# Thromboelastometry profile in critically ill patients: A single-center, retrospective, observational study

**DOI:** 10.1371/journal.pone.0192965

**Published:** 2018-02-20

**Authors:** Tomaz Crochemore, Thiago Domingos Corrêa, Marcus D. Lance, Cristina Solomon, Ary Serpa Neto, João Carlos de Campos Guerra, Priscila Scolmeister Lellis, Livia Muller Bernz, Natalia Nunes, Cassio Massashi Mancio, Ana Paula Hitomi Yokoyama, Eliézer Silva

**Affiliations:** 1 Hospital Israelita Albert Einstein, Intensive Care Unit, São Paulo, Brazil; 2 Hamad Medical Corporation | HMC · Anesthesiology, ICU and perioperative medicine –Doha/ Qatar; 3 Research & Development Department, Octapharma, Lachen, Switzerland; 4 Department of Anesthesiology, Perioperative Care and General Intensive Care, Paracelsus Medical University, Salzburg University Hospital, Salzburg, Austria; 5 Hospital Israelita Albert Einstein, Laboratory Center, São Paulo, Brazil; 6 Hospital Israelita Albert Einstein, Blood Bank, São Paulo, Brazil; Azienda Ospedaliero Universitaria Careggi, ITALY

## Abstract

**Background:**

Transfusion therapy is associated with increased morbidity, mortality and costs. Conventional coagulation tests (CCT) are weak bleeding predictors, poorly reflecting coagulation in vivo. Thromboelastometry (ROTEM) provides early identification of coagulation disorders and can guide transfusion therapy by goals, reducing blood components transfusion.

**Objective:**

The aim of this study is to describe coagulation profile of critically ill patients using ROTEM and evaluate the association between CCT and thromboelastometry.

**Methods:**

This is a retrospective, observational study conducted in medical-surgical intensive care unit (ICU). Adult patients (≥18 years) admitted to ICU between November 2012 and December 2014, in whom ROTEM analyses were performed for bleeding management were included in this study. The first ROTEM and CCT after ICU admission were recorded simultaneously. Additionally, we collected data on blood components transfusion and hemostatic agents immediately after laboratory tests results.

**Results:**

The study included 531 patients. Most ROTEM tests showed normal coagulation profile [INTEM (54.8%), EXTEM (54.1%) and FIBTEM (53.3%)] with divergent results in relation to CCT: low platelet count (51.8% in INTEM and 55.9% in EXTEM); prolonged aPTT (69.9% in INTEM and 63.7% in EXTEM) and higher INR (23.8% in INTEM and 27.4% in EXTEM). However 16,7% of patients with normocoagulability in ROTEM received platelet concentrates and 10% fresh frozen plasma.

**Conclusion:**

The predominant ROTEM profile observed in this sample of critically ill patients was normal. In contrast, CCT suggested coagulopathy leading to a possibly unnecessary allogenic blood component transfusion. ROTEM test may avoid inappropriate allogeneic blood products transfusion in these patients.

## Introduction

The hemostatic system, composed by soluble coagulation proteins, platelets, endothelium, natural anticoagulants, fibrinolytic system and their inhibitors, is driven by several regulatory mechanisms responsible for initiation, propagation, stabilization and clot lysis [1]. Countless diseases in intensive care unit (ICU) are associated with systemic inflammatory response syndrome (SIRS) and endothelial damage, which compromise the delicate and complex balance between anticoagulant and procoagulant systems [2]. As a result, clinical manifestations of varying degrees of hemorrhage or thrombosis may occur, impacting on patients outcomes [3].

Conventional coagulation tests (CCT) such as prothrombin time (PT) and activated partial thromboplastin time (aPTT), traditionally used to evaluate coagulation disorders, have limited accuracy to characterize the hemostatic profile and predict bleeding risk in critical ill patients [3, 4]. Moreover, CCT are unable to access clot strength and clot stability since such tests are read at the beginning of the fibrin polymerization process when only approximately 5% of thrombin generation occurred [5]. Furthermore, CCT are performed in plasma samples. Therefore, information concerning platelet-function, fibrinolysis and hypercoagulability is not provided [5, 6]. Finally, CCT results may take up to 60 minutes to be available, precluding a fast and dynamic coagulation evaluation at bedside [3, 6].

Rotational thromboelastometry (ROTEM) allows a dynamic evaluation of clot viscoelastic properties through graphic representation of clot formation, thrombin generation, fibrin polymerization and clot lysis [7]. ROTEM is performed in a whole blood sample. Therefore, its analysis takes into account the complex interactions between different blood cells and their biochemical characteristics, accessing blood hemostatic profile in real time at bedside [7].

Blood transfusion has been associated with increased morbidity, mortality, length of ICU and hospital stay and costs [8–10]. The implementation of thromboelastometry-driven transfusion algorithms has led to a significant reduction in blood components transfusion in different populations of perioperative in critically ill patients [11–13]. Therefore, thromboelastometry has been considered safer and more cost-effective than CCT for diagnosis and management of complex cases of coagulation disorders involving critically ill patients [14].

## Objectives

The main purpose of this study was to describe the coagulation profile of critically ill bleeding patients admitted to the ICU based on ROTEM and CCT. Additionally, we aimed to determine the frequency of allogeneic blood transfusion and hemostatic drugs administration in this population of critically ill patients.

## Methods

### Study design

This was a retrospective, single-center, observational study performed in a medical-surgical ICU in a tertiary care hospital. This study was approved by the Research Ethics Committee of Hospital Israelita Albert Einstein (CAAE: 37519814.0.0000.0071) and informed consent was waived.

### Participants

Adult patients (≥18 years) admitted to the ICU, in whom ROTEM analyses were performed for bleeding manageent between September 1, 2012 and September 30, 2014 were included in this study.

### Collected variables

Demographic data, comorbidities, admission type (medical or surgical), diagnosis at ICU admission, Simplified Acute Physiologic Score (SAPS) 3 [15], length of stay and mortality in ICU were collected. The first ROTEM (INTEM, EXTEM and FIBTEM) and CCT [platelets count (10^3^/mm^3^), plasma fibrinogen concentration (mg/dL), aPTT (sec), PT (sec) and INR], simultaneously collected during the ICU stay were retrieved. Finally, blood component transfusion [platelet concentrate, fresh frozen plasma (FFP) and cryoprecipitate] and hemostatic agents [fibrinogen concentrate, prothrombin complex concentrate (PCC) and tranexamic acid], which were administrated based on ROTEM and CCT analyses, were collected.

### Coagulation analysis

#### Rotational thromboelastometry

Rotational thromboelastometry (ROTEM®, TEM International GmbH, Munich, Germany) analyses were performed with EXTEM (extrinsic coagulation pathway assessment), INTEM (intrinsic coagulation pathway assessment) and FIBTEM (extrinsic coagulation pathway assessment with additional platelet inhibition using Cytochalasin D) tests according to the manufacturer’s instructions [16]. The following parameters were recorded during ROTEM analysis: clotting time [CT; seconds (sec)], which represents the beginning of the test until clot firmness of 2 mm; clot formation time (CFT; sec), which represents time between detection of a clot firmness of 2 and 20 mm; and maximum clot firmness (MCF; mm), which represents the greatest amplitude of thromboelastometric trace and reflects clot “strength” [3].

ROTEM tests were performed by laboratory technicians. Blood samples of approximately 3 ml were collected by venipuncture into a tube with citrate (3.2%; Sarsted®, Wedel, Germany). Blood samples were processed within a maximum period of two hours for ROTEM analysis. The analyses were performed by pipetting 340 μl of citrated whole blood and 20 μl of 0.2 M calcium chloride with specific activators into a cup [16]. There was no change in methodology for test performance nor test controls (Rotrol N and Rotrol P) throughout the study period.

Normal coagulation profile on the ROTEM was defined according to reference values for CT, CFT and MCF (INTEM CT: 100–240 sec, INTEM CFT: 30–110 sec, INTEM MCF: 50–72 mm; EXTEM CT: 38–79 sec, EXTEM CFT: 34–159 sec, EXTEM MCF: 50–72 mm; FIBTEM MCF: 9–25 mm) (3). Hypocoagulability in ROTEM was defined as prolongation of CT (INTEM CT >240 sec or EXTEM CT >79 sec) and/or CFT (INTEM CFT >110 sec or EXTEM CFT >159 sec) and/or MCF reduction (MCF INTEM or EXTEM MCF <50 mm or FIBTEM MCF <9 mm) (3). Hypercoagulability in ROTEM was defined as a reduction in clotting time (INTEM CT <100 sec or EXTEM CT <38 sec), or clot formation time (INTEM CFT <30 sec or EXTEM CFT <34 sec) and/or an increase in MCF (MCF INTEM or EXTEM MCF >72 mm or FIBTEM MCF >25 mm) [17].

#### Conventional coagulation tests

Plasma fibrinogen concentration [Clauss method (Fibrinogen STAGO, Diagnostica Stago, Asnieres-sur-Seine, France)], aPTT (STA cephascreen STAGO, Diagnostica Stago, Asnieres-sur-Seine, France), PT and INR (STA Neoplastine CI Plus, Diagnostica Stago, Asnieres-sur-Seine, France) were performed with blood samples of approximately 3 ml collected by venipuncture into a tube with citrate (3.2%; Sarsted®, Wedel, Germany). The controls used were STA COAG control N+P, device: STA R evolution and STA Compact for coagulometric mothodology. Platelet count was performed on plasma samples with ethylenediamine tetraacetic acid (EDTA-tube; 3.2%; Sarsted®, Wedel, Germany) with E Check XE control (XE 2100, Sysmex, São Paulo, Brazil).

Coagulopathy was defined according to changes in CCT as follows: thrombocytopenia <150 x10^3^/mm^3^, serum fibrinogen concentration <150 mg/dL or prolonged global coagulation time, such as INR >1.5 and TTPa >32 seconds [18].

#### Transfusion therapy

Transfusion practice for management of active bleeding at Hospital Israelita Albert Einstein by using allogenic blood components (FFP, platelets and cryoprecipitate), coagulation factor concentrates (fibrinogen concentrate and PCC) and hemostatic agents were performed according to CCT and physician discretion. FFP (10 mL/kg body weight) or PCC (25 UI/kg body weight) was administered if INR was >1.5. For prolonged aPTT >32 sec, FFP (10 mL/kg body weight) was administered. When serum fibrinogen was <150 mg/dL, either cryoprecipitate (1–2 units/10 kg of body weight) or fibrinogen concentrate (2 to 4g) was administered. Patients with platelet count <50 x10^3^/mm^3^ with active bleeding, or <20 x 10^3^/mm^3^ without active bleeding or <100 x 10^3^/mm^3^ in patients undergoing surgical procedures in central nervous system received transfusions of random platelets (1 unit/10 kg of body weight) or by apheresis (1 unit). Tranexamic acid was indicated when hyperfibrinolysis was suspected (20mg/Kg of body weight). In case of coagulation disorders, correction of hypothermia (axillary temperature ≥35°C), hypocalcemia (ionized calcium ≥1.14 mmol/L) and acidosis (pH ≥7.31) was recommended [7].

### Statistical analysis

Categorical variables were presented as absolute and relative frequencies. Quantitative variables were presented as average and standard deviation (SD) or median and interquartile range (IQR) when appropriate. The pattern of distribution of continuous variables was evaluated using the Kolmogorov-Smirnov test.

Comparisons between groups of patients with different coagulation profile (normal, hypocoagulability and hypercoagulability) according to ROTEM and CCT were performed. Proportions were compared using chi-square test or Fisher's exact test when assumptions for Chi-square use were violated. Continuous variables were compared using analysis of variance with one factor (ANOVA) followed by Tukey test. In case of non-normal distribution of study variables, Kruskal-Wallis test followed by Mann-Whitney U test were applied. The level of significance was adjusted according to Bonferroni correction after multiple comparisons.

Two-tailed tests were used, and when p value was <0.05, the test was considered statistically significant. SPSS™ (IBM™ Statistical Package for the Social Science version 21.0) was used for statistical analyses.

## Results

### Patients characteristics

Patients were more frequently male, with a median (IQR) age of 63 (53–74) years and SAPS III score of 47 (35–60) (Table 1). Approximately half of the patients were admitted to ICU due to medical reasons. Sepsis was the most common medical reason for ICU admission while the most common operative admission diagnoses were abdominal and cardiovascular surgery. The overall ICU mortality among studied patients was 24.7% (Table 1).

**Table 1:** Patients characteristics.

Characteristics	Values
Age, years	63 (52–74)
Gender, male	330/531 (62.1)
SAPS III score	47 (35–60)
Need of norepinephrine	276/531 (52.0)
Need of mechanical ventilation	260/531 (49.0)
Days on mechanical ventilation	1 (0–2)
Length of ICU stay,days	3 (2–8)
Mortality at ICU	131/531 (24.7)
Type of admission	
Clinical	281/531 (52.9)
Surgical	250/531 (47.1)
Comorbidities	
Systemic hypertension	249/531 (46.9)
Diabetes mellitus	146 /531 (27.4)
Immunossupression	136/531 (25.6)
Liver cirrhosis	92/531 (17.3)
Heart failure	89/531 (16.8)
Chronic renal failure on RRT	56/531 (10.5)
Smoking	51/531 (9.6)
Atrial fibrillation	48/531 (9.0)
Chronic renal failure	46/531 (8.7)
Myocardial infarction	38/531 (7.2)
Stroke	33/531 (6.2)
Alcoholism	27/531 (5.1)
Nonoperative admission diagnoses*	
Sepsis	124/281 (44.1)
Gastrointestinal	49/281 (17.4)
Cardiovascular	35/281 (12.5)
Respiratory	21/281 (7.5)
Neurological	17/281 (6.0)
Hematologic	17/281 (6.0)
Trauma	13/281 (4.6)
Renal	3/281 (1.1)
Metabolic	2/281 (0.7)
Operative admission diagnoses*	
Abdominal	91/250 (36.4)
Cardiovascular	86/250 (34.4)
Orthopedic	20/250 (8.0)
Urologic	18/250 (7.2)
Neurological	15/250 (6.0)
Obstetric Thoracic	11/250 (4.4) 7/250 (2.8%)
Trauma	2/250 (0.8)

Data presented as No./total No. (%) or median (IQR). SAPS: Simplified acute physiologic score, ICU: Intensive care unit, RRT: Renal replacement therapy.

* Admission diagnoses accordingly SAPS III score.

**Fig 1:**
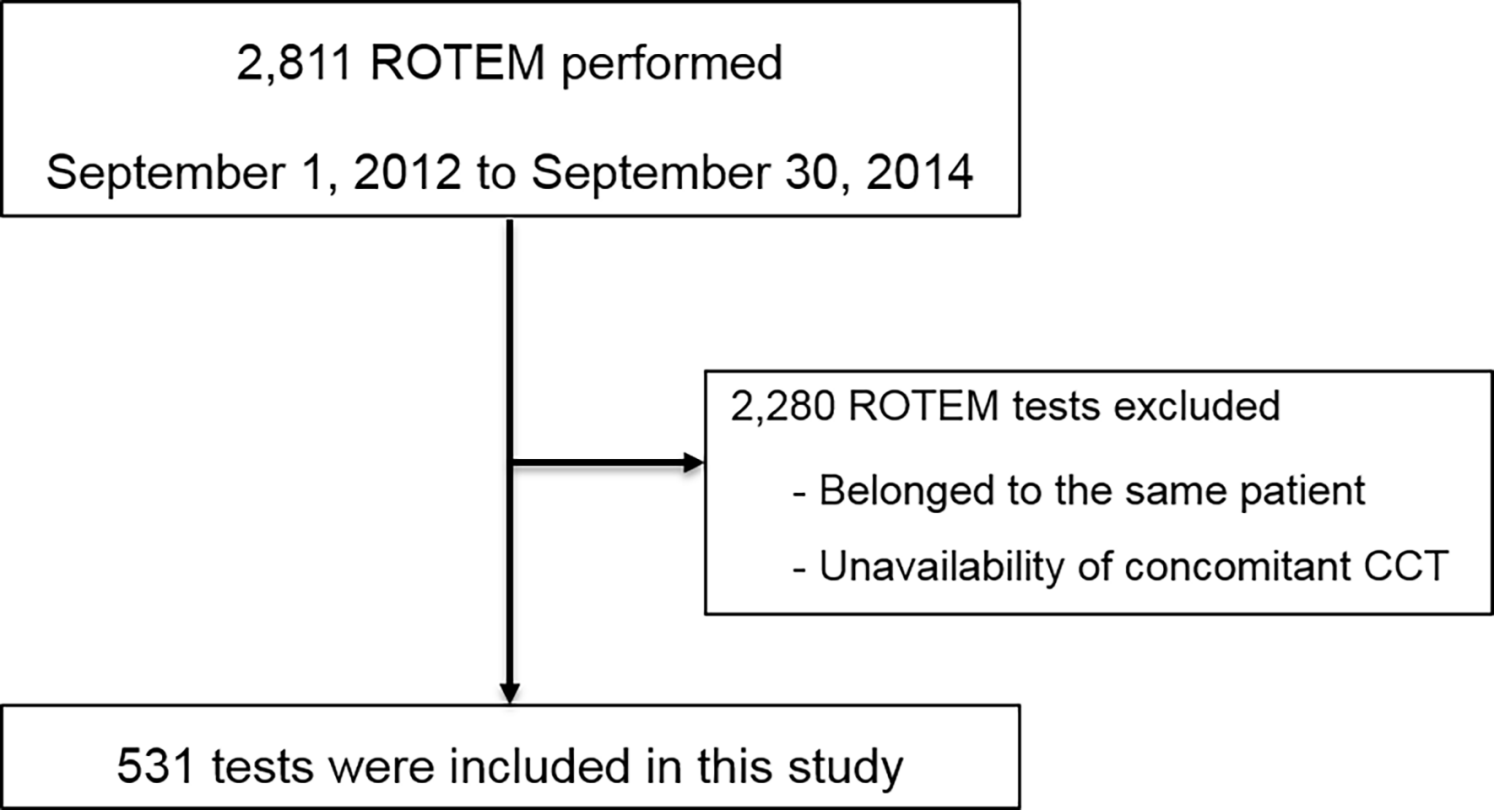
Study flowchart.

Between September 1, 2012 and September 30, 2014, 2811 ROTEM analyses were performed (Fig 1). After exclusion of 2,280 ROTEM analyses either for belonging to the same patient or for unavailability of concomitant CCT, 531 ROTEM analyses were included in this study (Fig 1).

### Coagulation profile

Results of CCT and ROTEM analyses are shown in Table 2. According to ROTEM, normal coagulation profile was observed, respectively for INTEM, EXTEM and FIBTEM, in 54.8% (193/352), 54.1% (179/331) and 53.3% (278/522) of patients (Table 3 and S1–S3 Tables). Among patients with altered ROTEM on INTEM or EXTEM, the majority presented a hypocoagulability (Table 3 and S1 and S3 Tables). Approximately half of the patients with altered ROTEM parameters on FIBTEM had a hypocoagulable profile [26.1% (136/522)] and half had [20.7% (108/522)] hypercoagulable profile (Table 3 and S3 Table). No cases of hyperfibrinolysis were observed in the studied patients.

**Table 2:** Coagulation profile of studied patients.

Characteristics	Values
Conventional coagulation tests	
INR	1.39 (1.18–2.02)
aPTT, sec	37.5 (32.0–48.8)
Platelets, x10^3^/mm^3^	111 (64–179)
Fibrinogen, mg/dl	287 (184–413)
Thromboelastometry	
INTEM CT, sec	190 (167–229)
INTEM CFT, sec	98 (63–175)
INTEM MCF, mm	56 (46–64)
EXTEM CT, sec	70 (59–85)
EXTEM CFT, sec	124 (81–201)
EXTEM MCF, mm	55 (46–65)
FIBTEM MCF, mm	15 (9–23)

Values represent median (IQR). INR: international normalized ratio, aPTT: activated thromboplastin time, CT: clotting time, CFT: clot formation time, MCF: maximum clot firmness.

**Table 3:** Conventional coagulation tests according to rotational thromboelastometry profile.

Characteristcs	Normal	Hypocoagulability	Hypercoagulability	P value*
INTEM	193/352 (54.8)	126/352 (35.8)	33/352 (9.4)	
Platelets <150 x10^3^/mm^3^	100/193 (51.8)	120/126 (95.2)	2/33 (6.1)	<0.001
INR >1.5	46/193 (23.8)	92/126 (73.0)	6/33 (18.2)	<0.001
aPTT >32 s	135/193 (69.9)	110/126 (87.3)	28/33 (84.8)	0.001
Fibrinogen <150 mg/dl	4/193 (2.1)	60/126 (47.6)	0/33 (0.0)	<0.001
EXTEM	179/331 (54.1)	133/331 (40.2)	19/331 (5.7)	
Platelets <150 x10^3^/mm^3^	100/179 (55.9)	128/133 (96.2)	3/19 (15.8)	<0.001
INR >1.5	49/179 (27.4)	100/133 (75.2)	6/19 (31.6)	<0.001
aPTT >32 s	114/179 (63.7)	120/133 (90.2)	15/19 (78.9)	<0.001
Fibrinogen <150 g/dl	7/179 (3.9)	65/133 (48.9)	0/19 (0.0)	<0.001
FIBTEM	278/522 (53.3)	136/522 (26.1)	108/522 (20.7)	
Platelets <150 x10^3^/mm^3^	155/278 (55.8)	123/136 (90.4)	53/108 (49.1)	<0.001
INR >1.5	80/278 (28.8)	104/136 (76.5)	30/108 (27.8)	<0.001
aPTT >32 s	184/278 (66.2)	117/136 (86.0)	90/108 (83.3)	<0.001
Fibrinogen <150 mg/dl	8/278 (2.9)	83/136 (61.0)	1/108 (0.9)	<0.001

Values represent No./total No. (%). INR: international normalized ratio and aPTT: activated partial thromboplastin time.

* p values were calculated with the use of Chi-square test or fisher exact test.

### Association between CCT and Rotem

Results comparing ROTEM parameters with CCT are shown in Table 3 and S3–S7 Tables. Among patients with normal coagulation profile on ROTEM, approximately half of them showed thrombocytopenia (platelets <150 x 10^3^/mm^3^) and prolonged aPTT (aPTT >32 s), and almost 25% of patients presented INR >1.5 (S3–S6 Tables). Abnormalities in CCT were even more pronounced in the presence of hypocoagulability since the majority of patients exhibited thrombocytopenia, aPTT prolongation and increased INR (S3–S6 Tables). Hypofibrinogenemia (fibrinogen <150 mg/dL) was found in approximately half of the patients with hypocoagulability profile on ROTEM (S3 and S7 Tables). Hypercoagulable patients according to ROTEM INTEM and EXTEM frequently exhibited prolonged aPTT and increased INR (Table 3 and S3 Table). Approximately half of hypercoagulable patients according to ROTEM FIBTEM had platelets count <150 x 10^3^/mm^3^ (Table 3 and S3 and S4 Tables).

### Administered treatment

Based on CCT results, more than one-third of bleeding patients received at least one type of blood component transfusion and approximately 34% of patients received coagulation factor concentrates or hemostatic drugs (Table 4 and S8 Table). The main blood component used was platelet concentrates (25.8%) followed by FFP (15.6%), while fibrinogen concentrate was the most frequently administered coagulation factor concentrate (Table 4 and S8 Table).

**Table 4:** Patients with allogeneic blood transfusion and hemostatic drugs administration based on conventional coagulation tests.

Characteristics	Values
Allogenic blood components	180/531 (33.9)
Platelets	137/531 (25.8)
Number of units, median (IQR)	1 (1–1)
Cryoprecipitate	35 / 531 (6.6)
Number of units, median (IQR)	1 (1–2)
Fresh frozen plasma	85/531 (15.6)
Number of units, median (IQR)	3 (2–5)
Hemostatic drugs	180/531 (33.9)
Fibrinogen concentrate	74/531 (13.9)
Prothrombin complex concentrate	48/531 (9.0)
Tranexamic acid	6/531 (1.1)

Values represent No./total No. (%) or median (IQR) when indicated.

### Administered treatment and Rotem profile

Approximately 22% (43/193) of patients with normal coagulation profile on INTEM and 19% (34/179) on EXTEM received platelet concentrates, FFP or cryoprecipitate while 24.5% (68/278) of patients with a normal coagulation profile on FIBTEM received platelets concentrate, FFP or cryoprecipitate (Table 5). Among hypocoagulable patients according to ROTEM, the most common administered blood component was platelet concentrate followed by FFP, while fibrinogen concentrate was the most frequently administered coagulation factor concentrate (Table 5).

**Table 5:** Transfusion therapy according to rotational thromboelastometry profile.

Characteristics	Normal	Hypocoagulability	Hypercoagulability	P value*
INTEM				
Platelets	33/193 (17.1)	59/126 (46.8)	0/33 (0.0)	<0.001
Fresh frozen plasma	22/193 (11.4)	37/126 (29.4)	3/33 (9.1)	<0.001
Cryoprecipitate	5/193 (2.6)	25/126 (19.8)	0/33 (0.0)	<0.001
PCC	9/193 (4.7)	17/126 (13.5)	3/33 (9.1)	0.016
Fibrinogen concentrate	10/193 (5.2)	43/126 (34.1)	1/33 (3.0)	<0.001
Tranexamic acid	3/193 (1.6)	0/126 (0.0)	0/33 (0.0)	0.465
EXTEM				
Platelets	26/179 (14.5)	59/133 (44.4)	2/19 (10.5)	<0.001
Fresh frozen plasma	18/179 (10.1)	31/133 (23.3)	2/19 (10.5)	0.005
Cryoprecipitate	5/179 (2.8)	14/133 (10.5)	0/19 (0.0)	0.012
PCC	14/179 (7.8)	21/133 (15.8)	0/19 (0.0)	0.027
Fibrinogen concentrate	7/179 (3.9)	49/133 (36.8)	0/19 (0.0)	<0.001
Tranexamic acid	3/179 (1.7)	1/133 (0.8)	1/19 (5.3)	0.299
FIBTEM				
Platelets	52/278 (18.7)	56/136 (41.2)	24/108 (22.2)	0.001
Fresh frozen plasma	32/278 (11.5)	36/136 (26.5)	12/108 (11.1)	0.001
Cryoprecipitate	6/278 (2.2)	26/136 (19.1)	0/108 (0.0)	0.001
PCC	17/278 (6.1)	23/136 (16.9)	5/108 (4.6)	0.001
Fibrinogen concentrate	21/278 (7.6)	48/136 (35.3)	1/108 (0.9)	0.001
Tranexamic acid	2/278 (0.7)	1/136 (0.7)	3/108 (2.8)	0.195

Values represent No./total No. (%). PCC: prothrombin complex concentrate.

* P values were calculated with the use of chi-square test or Fisher exact test.

## Discussion

The main finding of our study was that approximately half of bleeding patients with a normocoagulable state by ROTEM showed coagulopathy according to CCT, expressed predominantly by thrombocytopenia and prolongation of aPTT. Moreover, approximately one in five patients received platelet concentrates transfusion based on a low platelet count and more than 10% received FFP based on changes in CCT, yet both groups presented normal thromboelastometry.

Opposite to our findings, Halset and cols. demonstrated that more than 70% of adult patients admitted to ICU without bleeding or blood component transfusion in the preceding 24 hours exhibited a hypercoagulable profile on thromboelastography (TEG) [19]. Nevertheless, similarly to our findings, Ostrowski and cols. observed that approximately half of severe sepsis patients admitted to ICU presented normal coagulation, followed by hypocoagulability (30%) and hypercoagulable profile [20]. This disparity may be explained at least in part by different reference values of ROTEM used to define hypo- and hypercoagulability in such studies [21].

Contradictory findings about agreements between CCT and TEG/ROTEM to access the coagulation profile in critically ill patients have been reported [19, 22, 23]. While a poor agreement between CCT and TEG parameters to detect hypocoagulability in patients undergoing elective surgery has been reported by Ågren and cols. [22], good correlations between TEG and CCT have been described in elderly fractured patients [24] and in severe chronic liver patients [25].

Coagulopathy, defined by CCT, such as thrombocytopenia, prolongation of coagulation times (PT and aPTT) and reduction and/or dysfunction of plasma fibrinogen is common in critically ill patients [26]. The incidence of platelets <150 x 10^3^/mm^3^ and <50 x 10^3^/mm^3^ in critically ill patients can vary between 35–44% and 12–15%, respectively [27]. Prolongation of clotting times, PT or aPTT occurs in approximately 14–28% of critically ill patients [27]. The challenge at bedside is to distinguish between patients admitted to ICU presenting laboratory abnormalities without increased bleeding risk and those prone to developing severe bleeding as a result of coagulopathy, either spontaneously or due to surgical/invasive procedures.

Conventional coagulation tests only access 5% of thrombin generation process and represent weak predictors of bleeding in critically ill patients [28]. Conventional coagulation tests are performed on plasma samples in the absence of blood cells at a temperature of 37°C [29]. Moreover, CCT results can take up to 45 minutes to be available at bedside, making a prompt and precise approach of the critically ill patient with massive bleeding impossible [30].

Thromboelastometry allows an overall assessment of coagulation, including thrombin generation process, fibrin polymerization, clot strength and clot lysis in real time [31]. Furthermore, ROTEM allows early identification of specific coagulation disorders such as hypofibrinogenemia, hyperfibrinolysis and coagulation factors deficiency. Comparison of clot firmness in the EXTEM with clot firmness in FIBTEM allows the platelet component to be assessed [31]. Therefore, it has been demonstrated that thromboelastometry is an effective approach to guide transfusion therapy, according to individual needs [3]. Nevertheless, it is important to emphasize that platelets function in the strictest sense, effects of acetyl salicylic acid and adenosine‐diphosphate (ADP) receptor antagonists cannot be determined by the ROTEM [32,33].

Isolated evaluation of platelet count is unable to predict bleeding risk, since clot strength resulting from the interaction between platelets, plasma fibrinogen and factor XIII is not considered in CCT [33]. Thrombocytopenia may overestimate bleeding risk in critically ill patients, who often present high fibrinogen plasma levels, which is determining for clot firmness [34]. Furthermore, qualitative changes in fibrinogen function is not identified by the quantitative Clauss method, compromising bleeding risk analysis based on plasma fibrinogen levels [35]. Clot strength increases in a fibrinogen concentration-dependent manner, regardless of platelet count [34]. Conventional coagulation tests cannot address the relationship between platelets and fibrinogen. As a result, patients with a low platelet count might be assumed to have an increased risk of bleeding when accessed by CCT [36]. However, ROTEM allows evaluation of interaction between fibrinogen and platelets (maximum clot firmness), functionally (qualitatively) and quantitatively [32].

Many patients will exhibited a normocoagulable profile according to ROTEM, despite a low platelet count, possibly due to higher fibrinogen production secondary to an acute phase disease [37]. Even though the critically ill patient will develop thrombocytopenia usually on the 4^th^ day of hospitalization [38] for many different reasons (sepsis, heparin, drugs, and surgery), not necessarily all those patients will present a higher risk of bleeding and the need for platelet concentrates transfusion [18]. These patients often produce a higher amount of fibrinogen from the liver, compensating platelet deficiency and preserving clot strength [39, 40]. Thus, the practice of guiding blood components transfusion based only on changes in CCT may increase exposure of critically ill patients to different adverse events, with a negative impact on outcomes [12].

Our study has some limitations. First, this study was observational, retrospective, and single-center, which may compromise the external validity of our findings. Nevertheless, unlike other studies, our study involved a larger sample of critically ill patients with different medical or surgical diagnoses. Second, the use of anticoagulants and antiplatelet drugs were not available or recorded;thus our findings should be interpreted with caution. Due to the observational nature of this study, we could not determine if allogenic blood components, coagulation factor concentrates and hemostatic agents have been administered according to our hospital guidelines.

## Conclusion

Most of the critically ill patients admitted to ICU exhibited a normal coagulation profile according to ROTEM, although CCT suggested presence of coagulopathy. Transfusion therapy based on CCT led to a large number of patients receiving allogeneic blood transfusion, possibly unnecessarily. The use of ROTEM to identify the underlying coagulopathy and as a transfusion guide in this population of critically ill patients has the potential to avoid inappropriate allogeneic blood product transfusions.

## Supporting information

S1 Table Coagulation profile of studied patients accordingly to type of ICU admission. Data presented as median (interquartile range). ROTEM: rotational thromboelastometry, INR: international normalized ratio; aTTP: activated tromboplastin time; CT: clot time; CFT: clot formation time; MCF: maximum clot formation. p Values provide with Mann-Whitney U test.
(DOC)

S2 Table Rotational thromboelastometry profile. *p values provided with Kruskal-Wallis test. Pairwise comparisons significant at the 0.016 level: #: Normal vs. Hypo; &: Normal vs. Hyper and §: Hypo vs. Hyper. Values represent median (IQR).
(DOC)

S3 Table Conventional coagulation tests accordingly ROTEM profile. Values presented as median (interquartile range). INR: international normalized ratio and APTT: activated partial thromboplastin time. * P value provided by Kruskal-Wallis tests. Paired comparisons with significance level of 0016: #: Normal vs. Hypocoagulable; &: Normal vs. Hypercoagulable and §: Hypocoagulable vs. Hypercoagulable.
(DOC)

S4 Table Thromboelastometry profile according to platelets count. Data presented as no./total no. (%). p values provide with chi-square test.
(DOC)

S5 Table Comparisons between International Normalized Ratio (INR) and thromboelastometry profiles (ROTEM). Data presented as no./total no. (%). p values provide with chi-square.
(DOC)

S6 Table Comparisons between activated partial thromboplastin time (aPTT) and thromboelastometry profiles (ROTEM). Data presented as no./total no. (%). p values provide with chi-square.
(DOC)

S7 Table Comparisons between fibrinogen and thromboelastometry profiles (ROTEM). Data presented as no./total no. (%). p values provide with chi-square.
(DOC)

S8 Table Tranfusion therapy according to conventional coagulation tests. Data presented as no./total no. (%). p values provide with chi-square. INR: international normalized ratio, FFP: fresh frozen plasma, PCC: prothrombin complex concentrate, aPTT: activated partial thromboplastin time.
(DOC)

## Abbreviations

aPTT: activated partial thromboplastin time
CCT: conventional coagulation tests
CT: clot time
CFT: clot formation time
EXTEM: extrinsic thromboelastometry
FFP: fresh frozen plasma
ICU: intensive care unit
INR: international normalized ratio
INTEM: intrinsic thromboelastometry
MCF: maximum clot firmness
PCC: prothrombin complex concentrate
PT: prothrombin time
ROTEM: rotational thromboelastometry
SAPS III: Simplified Acute Physiologic Score III
TEG: thromelastrography
