# Supplementary material for: Thromboelastometry profile in critically ill patients: A single-center, retrospective, observational study
Source: PLoS One. 2018 Feb 20;13(2):e0192965. doi: 10.1371/journal.pone.0192965 (PMC5819777; doi:10.1371/journal.pone.0192965)
Supplement: S1 Table — Data presented as median (interquartile range). ROTEM: rotational thromboelastometry, INR: international normalized ratio; aTTP: activated tromboplastin time; CT: clot time; CFT: clot formation time; MCF: maximum clot formation. p Values provide with Mann-Whitney U test. (DOC) [file pone.0192965.s001.doc]

**S1 Table.** Coagulation profile of studied patients accordingly to type of ICU admission.

| **Parameters** | **Medical patients**  **281/531 (52.9%)** | **Surgical patients**  **250/531 (47.1%)** | **P value*** |
| --- | --- | --- | --- |
| **E Conventional Tests** |  |  |  |
| INR | 1.45 (1.18-2.40) | 1.32 (1.17-1.73) | 0.005 |
| aPTT, sec | 39.4 (33.2-50.3) | 35.7 (31.4-46.3) | 0.004 |
| Platelets, x 10^3^/mm^3^ | 100 (55-178) | 125 (78-180) | 0.004 |
| Fibrinogen, mg/dl | 322 (179-427) | 259 (188-377) | 0.091 |
| **ROTEM** |  |  |  |
| INTEM CT, s | 190 (170-237) | 192 (164-225) | 0.351 |
| INTEM CFT, s | 90 (58-189) | 102 (66-165) | 0.203 |
| INTEM MCF, mm | 58 (45-65) | 55 (47-63) | 0.478 |
| EXTEM CT, s | 72 (60-89) | 68 (58-81) | 0.046 |
| EXTEM CFT, s | 115 (73-208) | 130 (91-179) | 0.304 |
| EXTEM MCF, mm | 56 (43-65) | 55 (47-64) | 0.901 |
| FIBTEM MCF, mm | 17 (9-25) | 14 (9-20) | 0.001 |

Data presented as median (interquartile range). ROTEM: rotational thromboelastometry, INR: international normalized ratio; aTTP: activated tromboplastin time; CT: clot time; CFT: clot formation time; MCF: maximum clot formation. p Values provide with Mann-Whitney U test.
