# Supplementary material for: Thromboelastometry profile in critically ill patients: A single-center, retrospective, observational study
Source: PLoS One. 2018 Feb 20;13(2):e0192965. doi: 10.1371/journal.pone.0192965 (PMC5819777; doi:10.1371/journal.pone.0192965)
Supplement: S2 Table — *p values provided with Kruskal-Wallis test. Pairwise comparisons significant at the 0.016 level: #: Normal vs. Hypo; &: Normal vs. Hyper and §: Hypo vs. Hyper. Values represent median (IQR). (DOC) [file pone.0192965.s002.doc]

**S2 Table.** Rotational thromboelastometry profile.

| **Characteristics** | **Normal** | **Hypocoagulability** | **Hypercoagulability** | **P value*** |
| --- | --- | --- | --- | --- |
| **INTEM** |  |  |  |  |
| CT, sec | 175 (161-199)^#^ | 234 (198-287)^§^ | 179 (154-197) | <0.001 |
| CFT, sec | 74 (62-99)^#&^ | 219 (162-343)^§^ | 41 (35-44) | <0.001 |
| **MCF,** mm | 61 (56-65)^#&^ | 43 (34-47)^§^ | 76 (73-78) | <0.001 |
| **EXTEM** |  |  |  |  |
| CT , sec | 62 (57-72)^#^ | 85 (70-109)^§^ | 72 (60-77) | <0.001 |
| **CFT, sec** | 93 (73-117)^#&^ | 236 (177-366)^§^ | 46 (33-50) | <0.001 |
| **MCF, mm** | 62 (57-66)^#&^ | 42 (36-48)^§^ | 74 (72-78) | <0.001 |
| **FIBTEM** |  |  |  |  |
| **MCF, mm** | 16 (13-19)^#&^ | 7 (5-8)^§^ | 32 (27-38) | <0.001 |

*p values provided with Kruskal-Wallis test. Pairwise comparisons significant at the 0.016 level: #: Normal vs. Hypo; &: Normal vs. Hyper and §: Hypo vs. Hyper.

Values represent median (IQR).
