# Supplementary material for: Thromboelastometry profile in critically ill patients: A single-center, retrospective, observational study
Source: PLoS One. 2018 Feb 20;13(2):e0192965. doi: 10.1371/journal.pone.0192965 (PMC5819777; doi:10.1371/journal.pone.0192965)
Supplement: S3 Table — Values presented as median (interquartile range). INR: international normalized ratio and APTT: activated partial thromboplastin time. * P value provided by Kruskal-Wallis tests. Paired comparisons with significance level of 0016: #: Normal vs. Hypocoagulable; &: Normal vs. Hypercoagulable and §: Hypocoagulable vs. Hypercoagulable. (DOC) [file pone.0192965.s003.doc]

**S3 Table.** Conventional coagulation tests accordingly ROTEM profile.

| **Characteristcs** | **Normal** | **Hypocoagulability** | **Hypercoagulability** | **P value*** |
| --- | --- | --- | --- | --- |
| **INTEM** |  |  |  |  |
| Platelets <150 x10^3^/mm^3^ | 100/193 (51.8) | 120/126 (95.2) | 2/33 (6.1) | <0.001 |
| INR >1.5 | 46/193 (23.8) | 92/126 (73.0) | 6/33 (18.2) | <0.001 |
| aPTT >32 s | 135/193 (69.9) | 110/126 (87.3) | 28/33 (84.8) | 0.001 |
| Fibrinogen <150 mg/dl | 4/193 (2.1) | 60/126 (47.6) | 0/33 (0.0) | <0.001 |
| **EXTEM** |  |  |  |  |
| Platelets <150 x10^3^/mm^3^ | 100/179 (55.9) | 128/133 (96.2) | 3/19 (15.8) | <0.001 |
| INR >1.5 | 49/179 (27.4) | 100/133 (75.2) | 6/19 (31.6) | <0.001 |
| aPTT >32 s | 114/179 (63.7) | 120/133 (90.2) | 15/19 (78.9) | <0.001 |
| Fibrinogen <150 g/dl | 7/179 (3.9) | 65/133 (48.9) | 0/19 (0.0) | <0.001 |
| **FIBTEM** |  |  |  |  |
| Platelets <150 x10^3^/mm^3^ | 155/278 (55.8) | 123/136 (90.4) | 53/108 (49.1) | <0.001 |
| INR >1.5 | 80/278 (28.8) | 104/136 (76.5) | 30/108 (27.8) | <0.001 |
| aPTT >32 s | 184/278 (66.2) | 117/136 (86.0) | 90/108 (83.3) | <0.001 |
| Fibrinogen <150 mg/dl | 8/278 (2.9) | 83/136 (61.0) | 1/108 (0.9) | <0.001 |

Values presented as median (interquartile range). INR: international normalized ratio and APTT: activated partial thromboplastin time. * P value provided by Kruskal-Wallis tests. Paired comparisons with significance level of 0016: #: Normal vs. Hypocoagulable; &: Normal vs. Hypercoagulable and §: Hypocoagulable vs. Hypercoagulable.
