# Supplementary material for: Thromboelastometry profile in critically ill patients: A single-center, retrospective, observational study
Source: PLoS One. 2018 Feb 20;13(2):e0192965. doi: 10.1371/journal.pone.0192965 (PMC5819777; doi:10.1371/journal.pone.0192965)
Supplement: S4 Table — Data presented as no./total no. (%). p values provide with chi-square test. (DOC) [file pone.0192965.s004.doc]

**S4 Table.** Thromboelastometry profile according to platelets count.

| **Parameters** | **Platelets**  **<150** **x10^3^/mm^3^** | **Platelets**  **≥150** **x10^3^/mm^3^** | **P value** |
| --- | --- | --- | --- |
| **INTEM** |  |  | <0.001 |
| Normal | 100/222 (45.0) | 93/130 (71.5) |  |
| Hypocoagulability | 120/222 (54.1) | 6/130 (4.6) |  |
| Hypercoagulability | 2/222 (0.9) | 31/130 (23.8) |  |
| **EXTEM** |  |  | <0.001 |
| Normal | 100/231 (43.2) | 79/100 (79.0) |  |
| Hypocoagulability | 128/231 (55.4) | 5/100 (5.0) |  |
| Hypercoagulability | 3/231 (1.2) | 16/100 (16.0) |  |
| **FIBTEM** |  |  | <0.001 |
| Normal | 155/331 (46.8) | 123/191 (64.3) |  |
| Hypocoagulability | 123/331 (37.1) | 13/191 (6.8) |  |
| Hypercoagulability | 53/331(15.9) | 55/191 (28.0) |  |

Data presented as no./total no. (%). p values provide with chi-square test.
