# Supplementary material for: Thromboelastometry profile in critically ill patients: A single-center, retrospective, observational study
Source: PLoS One. 2018 Feb 20;13(2):e0192965. doi: 10.1371/journal.pone.0192965 (PMC5819777; doi:10.1371/journal.pone.0192965)
Supplement: S5 Table — Data presented as no./total no. (%). p values provide with chi-square. (DOC) [file pone.0192965.s005.doc]

**S5 Table.** Comparisons between International Normalized Ratio (INR) and thromboelastometry profiles (ROTEM).

| **Parameters** | **INR >1.5** | **INR ≤1.5** | **P Value** |
| --- | --- | --- | --- |
| **INTEM** |  |  | <0.001 |
| Normal | 46/144 (31.9) | 147/208 (70.7) |  |
| Hypocoagulability | 92/144 (63.9) | 34/208 (16.3) |  |
| Hypercoagulability | 6/144 (4.2) | 27/208 (13.0) |  |
| **EXTEM** |  |  | <0.001 |
| Normal | 49/155 (31.6) | 130/176 (73.9) |  |
| Hypocoagulability | 100/155 (64.5) | 33/176 (18.8) |  |
| Hypercoagulability | 6/155 (3.9) | 13/176 (7.4) |  |
| **FIBTEM** |  |  | <0.001 |
| Normal | 80/214 (37.4) | 198/308 (64.3) |  |
| Hypocoagulability | 104/214 (48.6) | 32/308 (10.4) |  |
| Hypercoagulability | 30/214 (14.0) | 78/308 (25.3) |  |

Data presented as no./total no. (%). p values provide with chi-square.
