# Supplementary material for: Thromboelastometry profile in critically ill patients: A single-center, retrospective, observational study
Source: PLoS One. 2018 Feb 20;13(2):e0192965. doi: 10.1371/journal.pone.0192965 (PMC5819777; doi:10.1371/journal.pone.0192965)
Supplement: S6 Table — Data presented as no./total no. (%). p values provide with chi-square. (DOC) [file pone.0192965.s006.doc]

**S6 Table.** Comparisons between activated partial thromboplastin time (aPTT) and thromboelastometry profiles (ROTEM).

| **Parameters** | **aPTT >32 s** | **aPTT ≤32 s** | **P value** |
| --- | --- | --- | --- |
| **INTEM** |  |  | <0.001 |
| Normal | 135/273 (49.5) | 58/79 (73.4) |  |
| Hypocoagulability | 110/273 (40.3) | 16/79 (20.3) |  |
| Hypercoagulability | 28/273(10.3) | 5/79 (6.3) |  |
| **EXTEM** |  |  | <0.001 |
| Normal | 114/249 (45.8) | 65/82 (79.3) |  |
| Hypocoagulability | 120/249 (48.2) | 13/82 (15.9) |  |
| Hypercoagulability | 15/249 (6.0) | 4/82 (4.9) |  |
| **FIBTEM** |  |  | <0.001 |
| Normal | 184/391 (47.1) | 94/131 (71.8) |  |
| Hypocoagulability | 117/391 (29.9) | 19/131 (14.5) |  |
| Hypercoagulability | 90/391 (23.0) | 18/131 (13.7) |  |

Data presented as no./total no. (%). p values provide with chi-square.
