# Supplementary material for: Thromboelastometry profile in critically ill patients: A single-center, retrospective, observational study
Source: PLoS One. 2018 Feb 20;13(2):e0192965. doi: 10.1371/journal.pone.0192965 (PMC5819777; doi:10.1371/journal.pone.0192965)
Supplement: S7 Table — Data presented as no./total no. (%). p values provide with chi-square. (DOC) [file pone.0192965.s007.doc]

**S7 Table.** Comparisons between fibrinogen and thromboelastometry profiles (ROTEM).

| **Parameters** | **Fibrinogen**  **<150 mg/dL** | **Fibrinogen**  **≥150 mg/dL** | **P Value** |
| --- | --- | --- | --- |
| **FIBTEM** |  |  | <0.001 |
| Normal | 8/92 (8.7) | 270/430 (62.8) |  |
| Hypocoagulability | 83/92 (90.2) | 53/430 (12.3) |  |
| Hypercoagulability | 1/92 (1.1) | 107/430 (24.9) |  |

Data presented as no./total no. (%). p values provide with chi-square.
