# Supplementary material for: Thromboelastometry profile in critically ill patients: A single-center, retrospective, observational study
Source: PLoS One. 2018 Feb 20;13(2):e0192965. doi: 10.1371/journal.pone.0192965 (PMC5819777; doi:10.1371/journal.pone.0192965)
Supplement: S8 Table — Data presented as no./total no. (%). p values provide with chi-square. INR: international normalized ratio, FFP: fresh frozen plasma, PCC: prothrombin complex concentrate, aPTT: activated partial thromboplastin time. (DOC) [file pone.0192965.s008.doc]

**S8 Table.** Tranfusion therapy according to conventional coagulation tests.

| **Characteristcs** | **Normal** | **Hypocoagulable** | **P value** |
| --- | --- | --- | --- |
| INR | 322/531 (60.0) | 209/531 (39.3) |  |
| FFP | 34/322 (10.6) | 49/209 (23.4) | <0.001 |
| PCC | 14/322 (4.3) | 34/209 (16.3) | <0.001 |
| aPTT | 90/531 (16.9) | 441/531 (83.0) |  |
| FFP | 6/90 (6.7) | 77/441 (17.5) | <0.001 |
| Platelets | 440/531 (82.8) | 91/531 (17.1) |  |
| Platelets concentrate | 83/440 (18.9) | 54/91 (59.3) | <0.001 |
| Serum fibrinogen | 433/531 (81.5) | 98/531 (18.4) |  |
| Fibrinogen concentrate | 32/433 (7.4) | 42/98 (42.9) | <0.001 |
| Cryoprecipitate | 9/433 (2.1) | 26/98 (26.5) | <0.001 |

Data presented as no./total no. (%). p values provide with chi-square. INR: international normalized ratio, FFP: fresh frozen plasma, PCC: prothrombin complex concentrate, aPTT: activated partial thromboplastin time.
